# Cofilin1 is involved in hypertension-induced renal damage via the regulation of NF-κB in renal tubular epithelial cells

**DOI:** 10.1186/s12967-015-0685-8

**Published:** 2015-10-08

**Authors:** Quan-zhen Wang, Hai-qing Gao, Ying Liang, Jun Zhang, Jian Wang, Jie Qiu

**Affiliations:** Department of Geriatric Cardiology, Qilu Hospital of Shandong University, 107 Wenhua Xi Rd, 250012 Jinan, People’s Republic of China; Department of Geriatric Cardiology, Qianfuoshan Hospital of Shandong Province, 16766 Jingshi Rd, 250000 Jinan, People’s Republic of China

**Keywords:** Cofilin1, Hypertension, Renal damage, Cytoskeleton, NF-κB

## Abstract

**Background:**

Inflammation mediated by nuclear factor-κB (NF-κB) plays a critical role in the pathogenesis of hypertensive nephropathy (HN). Cytoskeletal remodelling is necessary for the activation of NF-κB. An actin-binding protein, cofilin-1 promotes dynamic alterations to the cytoskeleton by severing actin filaments. However, whether cofilin1 modulates NF-κB activity via cytoskeletal remodelling in the setting of hypertensive renal damage and what mechanisms underlie this phenomenon, remain unknown.

**Methods:**

Twenty-one-week old spontaneously hypertensive rats (SHRs) were treated with an antioxidant (100 or 250 mg kg^−1^ day^−1^), grape seed proanthocyanidins extract (GSPE), for 22 weeks. Twenty-four-hour urinary protein, serum creatinine and urea nitrogen levels were measured. Haematoxylin and eosin (HE) staining was performed, and the expression levels of renal cortex cofilin1, monocyte chemotactic protein 1 (MCP1), interleukin-1β (IL1β) and NF-κB were evaluated via either Western blotting or immunohistochemistry. In vitro, human proximal renal tubular epithelial cells (HK-2 cells) were pre-incubated either with or without GSPE and subsequently treated with angiotensinII (AngII). Furthermore, a lentiviral shRNA-vector was utilized to knockdown cofilin1 expression in the HK-2 cells, which were stimulated with AngII. Actin filaments, NF-κB activity and several downstream inflammatory factors, including MCP1 and IL-1β, were investigated.

**Results:**

In addition to elevated blood pressure and 24 h urinary protein levels, NF-κB activity and the expression levels of MCP1 and IL-1β were significantly increased, resulting in tubulointerstitial inflammatory infiltration in SHRs. The phosphorylation (inactivation) of cofilin1 was increased in the kidneys of the SHRs. In vitro, AngII stimulation resulted in the phosphorylation of cofilin1, the formation of actin stress fibres and nuclear translocation of NF-κB p65 in the HK2 cells. Both GSPE pretreatment and the shRNA knockdown of cofilin1 inhibited Rel/p65 nuclear translocation, as well as the expression of both MCP-1 and IL-1β in the AngII-induced HK2 cells.

**Conclusion:**

These results demonstrate that cofilin1 is involved in hypertensive nephropathy by modulating the nuclear translocation of NF-κB and the expression of its downstream inflammatory factors in renal tubular epithelial cells.

**Electronic supplementary material:**

The online version of this article (doi:10.1186/s12967-015-0685-8) contains supplementary material, which is available to authorized users.

## Background

Hypertension represents one of the most important risk factors for end-stage kidney disease in America, second only to diabetes [1]. Recent studies have demonstrated that hypertensive renal damage is associated with oxidative stress and inflammation [2, 3].

A ubiquitously inflammatory transcription factor, nuclear factor κB (NF-κB) plays an important role in the pathogenesis of hypertensive nephropathy (HN) [4, 5]. Inactive NF-κB is sequestered within the cytoplasm and bound by the members of the IκB family of inhibitor proteins. The various stimuli that activate NF-κB cause the phosphorylation of IκB, which is followed by its ubiquitination and subsequent degradation. Finally, NF-κB migrates to the nucleus and activates the transcription of several target genes, including monocyte chemotactic protein 1(MCP1) and Interleukin-1β (IL1β). These genes induce persistent kidney inflammation. The translocation of RelA/p65 (an NF-κB subunit) to the nucleus requires dynamic alterations in the actin cytoskeleton; interfering with these alterations inhibits the nuclear accumulation of RelA/p65 [6]. Therefore, actin cytoskeletal remodelling may influence NF-κB activity and participate in renal inflammation.

Cofilin1 is an actin-binding protein that plays an essential role in the regulation of actin filament dynamics and reorganization by stimulating the severance and depolymerization of actin filaments [7], and has been identified as a critical determinant of thrombin-induced RelA/p65 nuclear translocation via modifications of actin dynamics [8]. Additionally, cofilin-1 serves as an important ‘‘functional node’’ within podocytes [9]. However, whether cofilin1 regulates actin filaments and Rel/p65 nuclear translocation in renal tubular epithelial cells remains unclear.

Antioxidant-enriched diets reduce renal interstitial inflammation and improve hypertension in spontaneously hypertensive rats (SHRs) [10]. Grapes are one of the most widely consumed fruits worldwide and are rich in polyphenols. Grape polyphenols, particularly anthocyanins and flavonols, inhibit proinflammatory cytokines and the metabolic endotoxin-triggered activation of inflammatory MEK/MAPK, NF-κB, and AP-1 signalling, which increases inflammatory genes expression (e.g., TNF-α, IL-6, IL-8, IL-1β, and MCP-1) [11]. Gravinol, a proanthocyanidin found in grape seeds, inhibits NF-κB nuclear translocation in high glucose-treated renal tubular epithelial cells [12].

Accordingly, the present study was performed to investigate whether cofilin1 is involved in hypertensive renal inflammation and to determine the intracellular mechanisms underlying said involvement. We selected grape seed proanthocyanidins extract (GSPE) as a control.

## Methods

### Animals

Twenty-week-old male spontaneous hypertensive rats (SHRs) and Wistar-Kyoto rats (WKYs) were obtained from the Vital River Laboratory Animal Co. Ltd (Beijing, China). The animals were housed under standard conditions and 12 h light/dark cycles. They were given free access to tap water and standard rat chow. Following 1 week of acclimation, the rats were randomly assigned to the following four groups (n = 10 per group): SHR-C (a SHR control group administered 1 mL 0.9 % sodium chloride orally), WKY-C (a WKY control group administered 1 mL 0.9 % sodium chloride orally), SHR-H (SHRs administered GSPE at a dose of 250 mg kg^−1^ day^−1^), SHR-L (SHRs administered GSPE at a dose of 100 mg kg^−1^ day^−1^). The animals were subjected to drug treatments as indicated above via oral gavage for 22 weeks. GSPE was provided by Jianfeng Inc. (Tianjin, China) and contained ≥95.0 % proanthocyanidins. The components of GSPE were analysed via high-performance liquid chromatography (HPLC)-UV. All animals were cared for in accordance with the National Institute of Health Guide for the Care and Use of Laboratory Animals, with the approval of the Scientific Investigation Ethics Committee of Qilu Hospital, Shandong University.

### Systolic blood pressure measurements

Tail systolic blood pressures (SBP) were measured weekly until the end of the experiment (22 times in total) in conscious rats using a Softron tail-cuff BP-2006A non-invasive sphygmomanometer (Softron Incorporated, Beijing, China).

### Urine and serum measurement

The rats were housed in individual metabolic cages for the collection of urine at 2-week intervals. The urine was centrifuged and stored at −80 °C. Treatment was continued for 22 weeks, at which time the rats were weighed and anaesthetized using chloral hydrate. Blood was collected from the abdominal aorta and centrifuged to obtain serum, which was stored at −80 °C. Twenty-four-hour urinary protein, serum creatinine and urea nitrogen were determined using an automatic biochemical analyser and served as indices of renal injury.

### Histology

Immediately following anaesthesia, the kidneys were removed. The left kidney was processed for histology and immunostaining, whereas the right kidney was used for Western blotting. The kidneys from ten rats per group were fixed in 4 % neutral buffered formalin and subsequently embedded in paraffin. The 4 µm sections of paraffin embedded tissues were subsequently stained with haematoxylin and eosin (HE) and analysed under a light microscope by two investigators blinded to the treatments. To assess the degree of tubulointerstitial inflammation, sections from each animal were evaluated. In each section, 10 randomly selected observation fields were examined. The number of inflammatory cells in the tubulointerstitium was counted under high-power fields (40×) using a 0.0625-mm^2^ graticule fitted to the eyepiece of the microscope as previously described [13].

### Immunohistochemical analysis

Following deparaffinization, hydration, blockage of endogenous peroxidase and antigen retrieval routinely, the renal sections were incubated with primary antibodies for MCP1, IL-1β (1:200, Abcam, Shanghai, China) or NF-κB(1:300, CST, USA) at 4 °C overnight. The slides were subsequently incubated with goat anti-rabbit IgG (ZSGB-BIO, Beijing, China) at 37 °C for 30 min, followed by staining with diaminobenzidine. A negative control was prepared by omitting the primary antibody (data were not shown). The stained tissue slides were observed under an Olympus microscope (model IX81, Japanese). The expression levels of MCP-1, IL-1β and NF-κB p65 in the cortical tubulointerstitium (cross section of the renal cortex) were determined using quantitative Image-Pro plus software as described previously [14].

### ELISA

The protein levels of IL-1β and MCP1 in the renal tissues were measured using an ELISA kit (EBioscience, USA) according to the manufacturer’s instructions. Briefly, protein samples from four groups (n = 7) were homogenized with ice cold extraction buffer (1 mol L^−1^ HCl and neutralized with 1.2 mol L^−1^ NaOH/0.5 mol L^−1^ HEPES). The homogenate was centrifuged at 12,000*g* for 15 min at 4 °C. The supernatant was used to assay the amounts of IL1β and MCP1. Absorbance was determined at 450 nm using an ELISA plate reader (INIFINITE M200, TECAN, Switzerland).

### Cell culture and treatment

Human renal proximal tubular cells (HK-2) were purchased from the American Type Culture Collection (Manassas, VA, USA) and maintained in DMEM/F12 (Gibco, Carlsbad, USA) medium supplemented with 10 % foetal bovine serum (FBS, Gibco, Carlsbad, USA), 100 U mL^−1^ penicillin and 100 µg mL^−1^ streptomycin (Solarbio, Beijing, China). These cells were routinely cultured at 37 °C in a humidified atmosphere of 95 % air-5 % CO_2_ and nourished at intervals of 2–3 days. Subconfluent HK2 cells were preincubated in either the presence or the absence of GSPE (50 µg mL^−1^) for 12 h before being stimulated either with or without AngII (10^−6^ mol L^−1^, Sigma, Shanghai, China) for 12 h. GSPE was dissolved in DMSO and diluted so that the final concentration of DMSO was <0.1 %.

### Knockdown of cofilin-1

Lentiviral-shRNA specific for interfering cofilin-1 expression, recombinant lentiviral Lent/Cof and a nonspecific lentiviral control were obtained from GeneChem (GeneChem, Shanghai, China). These lentiviral expression vectors contained the eGFP reporter gene (enhanced green fluorescent protein). The cells were transfected with lentiviral suspension using transfection reagent according to the manufacturer’s recommendations. Following 72–96 h, transfection efficiency was measured by testing the expression ratio of eGFP via fluorescence microscopy. Moreover, the knockdown of cofilin1 was evaluated via Western blotting.

### Luciferase reporter gene assay

The HK2 cells were seeded in 24-well plates and grown overnight to 80–90 % confluence; 0.8 µg NF-κB of luciferase reporter (pNF-κB-TA-luc) and the internal control plasmid pGL6-TA (Byotime, Shanghai, China) were transfected into cells via Lipofectamine™ 2000 and placed in fresh medium after 6 h. Following transfection for 30–48 h, the cells were stimulated with 10^−6^ mol L^−1^ of AngII. Twelve hours later, the cells were harvested to quantify luciferase activity using a dual luciferase reporter assay kit (Beyotime, Shanghai, China) according to the manufacture’s protocol. Regarding the experiments investigating the effects of cofilin1 knockdown on NF-κB activity, the cells were first transfected with either recombinant lentiviral Lent/Cof or a nonspecific lentiviral control. Following passage, the cells were again transfected via pNF-κB-TA-luc and analysed as described above.

### Immunofluorescence

Cells from different groups were grown on coverslips and washed three times with phosphate-buffered saline (PBS), fixed in 4 % paraformaldehyde for 20 min and permeabilized with 0.2 % Triton X-100 for 10 min at room temperature. Following additional washes, the cells were incubated in blocking solution (1 % bovine serum albumin/PBS) for 30 min at room temperature in order to remove non-specifically bound antibodies. To localize the F-actin filaments, the cells were incubated with 5 µg mL^−1^ rhodamine-phalloidin (Sigma, USA) for 30 min at 37 °C in a humid chamber. RelA/p65 was detected using a rabbit monoclonal anti-RelA/p65 antibody (1:100, CST, USA) overnight at 4 °C. The cells were washed again and incubated in the dark at room temperature for 1 h with a secondary antibody [1:500, Cy3-labeled goat anti-rabbit IgG (H + L), Beyotime, Shanghai, China]. Following washing with PBS in the dark, DAPI was used to counterstain the nucleus for 5 min (in the dark at room temperature). Images were obtained using an Olympus microscope (model IX-81; Japanese).

### Western blotting

The nuclear and cytoplasmic proteins of the HK-2 cells were extracted using a commercially available assay kit (Byotime, Shanghai, China). The total proteins of the HK2 cells and renal cortex were extracted as published previously [15, 16]. The protein concentrations were determined using a BCA assay kit (Byotime, Shanghai, China) as instructed. The proteins (20–80 µg) were separated via SDS-PAGE and transferred onto PVDF membranes before being blocked in 5 % no-fat milk in TBST (0.1 % Tween20 in Tris-buffered saline) and incubated with dilutions of anti-cofilin1 (1:1000, CST, USA), anti-phosphate-cofilin1 (1:500, CST, USA), anti-RelA/p65 (1:1000, CST, USA), anti-IκB-α (1:500, Abcam, Shanghai), anti-MCP1 (1:500, Abcam, Shanghai), anti-IL-1β (1:1000, Abcam, Shanghai) and anti-GAPDH (1:1000, SAB, USA) overnight at 4 °C. Following incubation for 2 h at room temperature with secondary antibodies (1:5000, ZSGB-BIO, Beijing, China), the target bands were visualized using an enhanced chemiluminescence detection system (Image Quant LAS 4000 mini, GE, USA) and analysed densitometrically using Quantity one software (Bio-Rad, USA).

### Statistical analysis

All experiments were performed in triplicate. The data were analysed using SPSS 13.0 statistical software. All values are presented as means ± standard deviations (SDs). The one-way ANOVA post hoc multiple comparisons LSD and t test were used for statistical comparison between the groups and within the groups. P < 0.05 (two-tailed) was indicative of a statistically significant difference.

## Results

### Renal parameters and blood pressure

Urine protein over 24 h was significantly increased in the SHRs compared with the age matched WKY (P < 0.01, Table 1). Following 22 weeks of therapy with GSPE (100 or 50 mg kg^−1^ day^−1^), the SHRs exhibited significantly reduced urinary protein levels. However, there were no significant differences observed with respect to blood urea nitrogen, serum and creatinine across the groups in either the WKY rats or the SHRs, either before or after treatment. Compared with the beginning of our experiment, the SBP level of the 21-week-old SHRs was significantly higher than that of WKY rats (P < 0.01, Additional file 1: Figure S1) and gradually increased as time passed. However, GSPE treatment failed to attenuate the blood pressure increases in SHRs in the present study (P > 0.05, Additional file 1: Figure S1).

**Table 1:** Clinical characteristics of WKYs and SHRs following 22-week treatment

Parameters	WKY-C (n = 10)	SHR-C (n = 10)	SHR-H (n = 10)	SHR-L (n = 10)
Weight (g)	350.55 ± 17.77	345.67 ± 11.63	344.89 ± 15.67	354.63 ± 11.64
BUN (mM)	9.19 ± 2.58	9.21 ± 2.13	9.16 ± 1.36	10.20 ± 1.63
Cr (uM)	32.57 ± 4.65	38.50 ± 17.17	30.00 ± 5.86	34.63 ± 10.42
UP (mg/24 h)	14.47 ± 5.45	39.14 ± 12.91**	23.79 ± 6.26**^##^	30.16 ± 5.43**^#^

Data are presented as mean ± SD, n = 10

*BUN* blood urea nitrogen, *Cr* serum creatinine, *UP 24* *h* urine protein for 24 h

* P < 0.05, ** P < 0.01 vs. WKY-C group

^#^ P < 0.05, ^##^ P < 0.05 vs. SHR-C group

### Renal histopathology and inflammatory cytokines

**Fig. 1:**
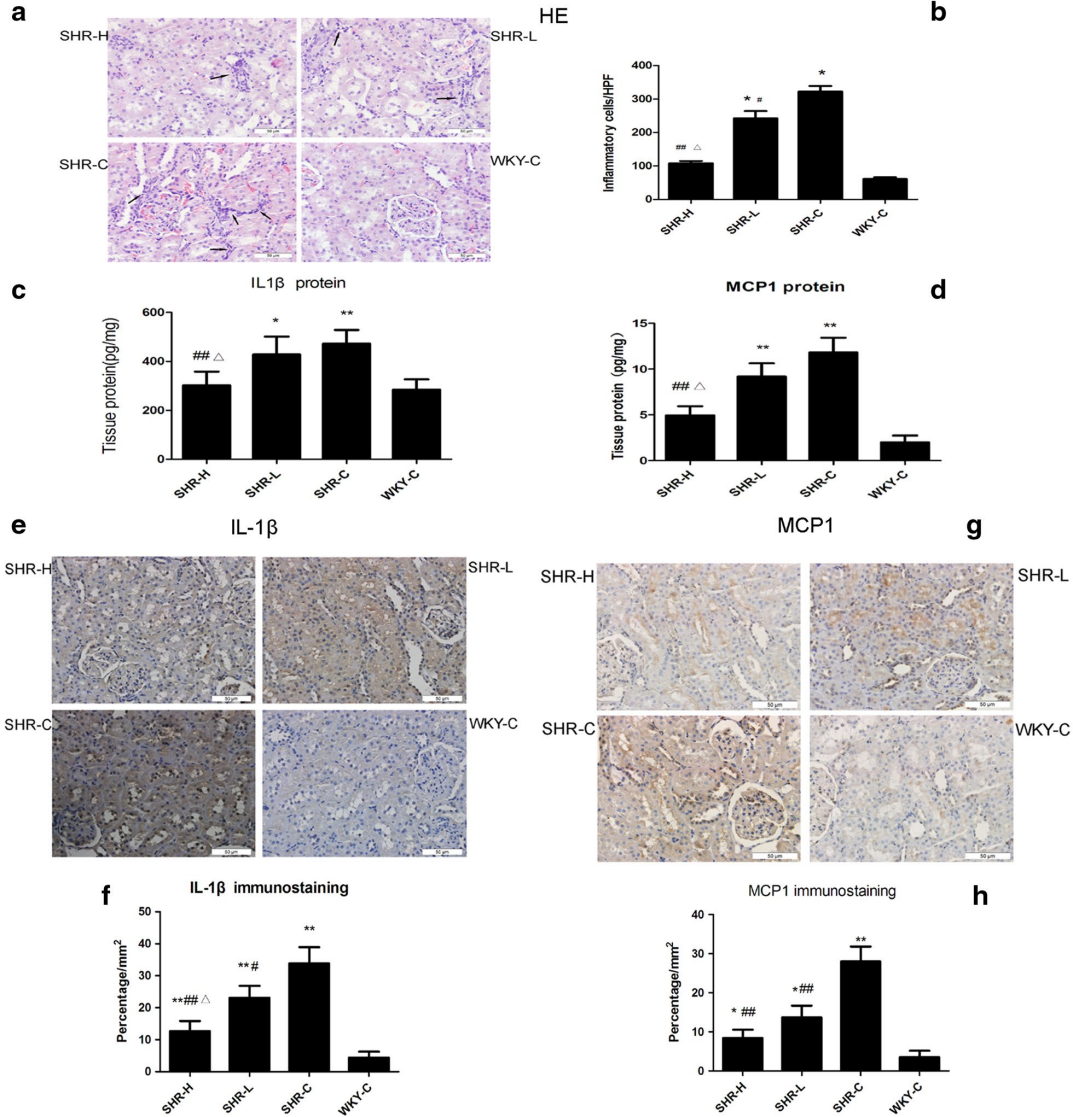
Renal inflammatory damage in hypertensive rats. **a**, **b** Tubulointerstitial inflammatory injury in the kidneys of SHRs and WKYs. **a** Haematoxylin and eosin (HE) staining of tubulointerstitial histopathology. The *arrows* indicate inflammatory cells. **b** The number of inflammatory cells (including monocytes, lymphocytes, macrophages, neutrophils) in tubulointerstitial areas of different HPFs (high-powered fields). **c**, **d** The protein expressions of IL-1β and MCP1 in rat kidneys were detected by ELISA. Results are expressed as pg/mg of total protein. **e**, **f** Renal sections show immunohistochemical staining of IL-1β, *bar charts* show quantification of IL-1β areas in each group. **g**, **h** Renal sections show immunohistochemical staining of MCP1, *bar charts* show quantification of MCP1 areas in each group. Data are presented as mean ± SD, n = 7 or 10. *P < 0.05, **P < 0.01 vs. WKY-C, ^#^P < 0.05, ^##^P < 0.01 vs. SHR-C and ΔP < 0.05 vs. SHR-L by ANOVA. Original magnification ×400, *Scar bar* represents 50 μm

Pathological changes were observed via HE staining and immunohistochemistry (Fig. 1a, e, g). A quantitative analysis demonstrated significant interstitial inflammatory infiltration (including neutrophils, lymphocytes and monocytes) in the kidneys of the SHR-L and SHR-C groups compared with the SHR-H and WKY-C rats. GSPE administration significantly alleviated the inflammatory infiltration in the renal interstitial. Additionally, the protein levels of both MCP1 and IL-1β in the kidneys of the SHRs increased compared with the WKY group. Following GSPE treatment, the upregulated MCP1 and IL1β protein levels were partially decreased in the SHRs (P < 0.05, Fig. 1c–h).

### Renal NF-κB and cofilin1 activity

**Fig. 2:**
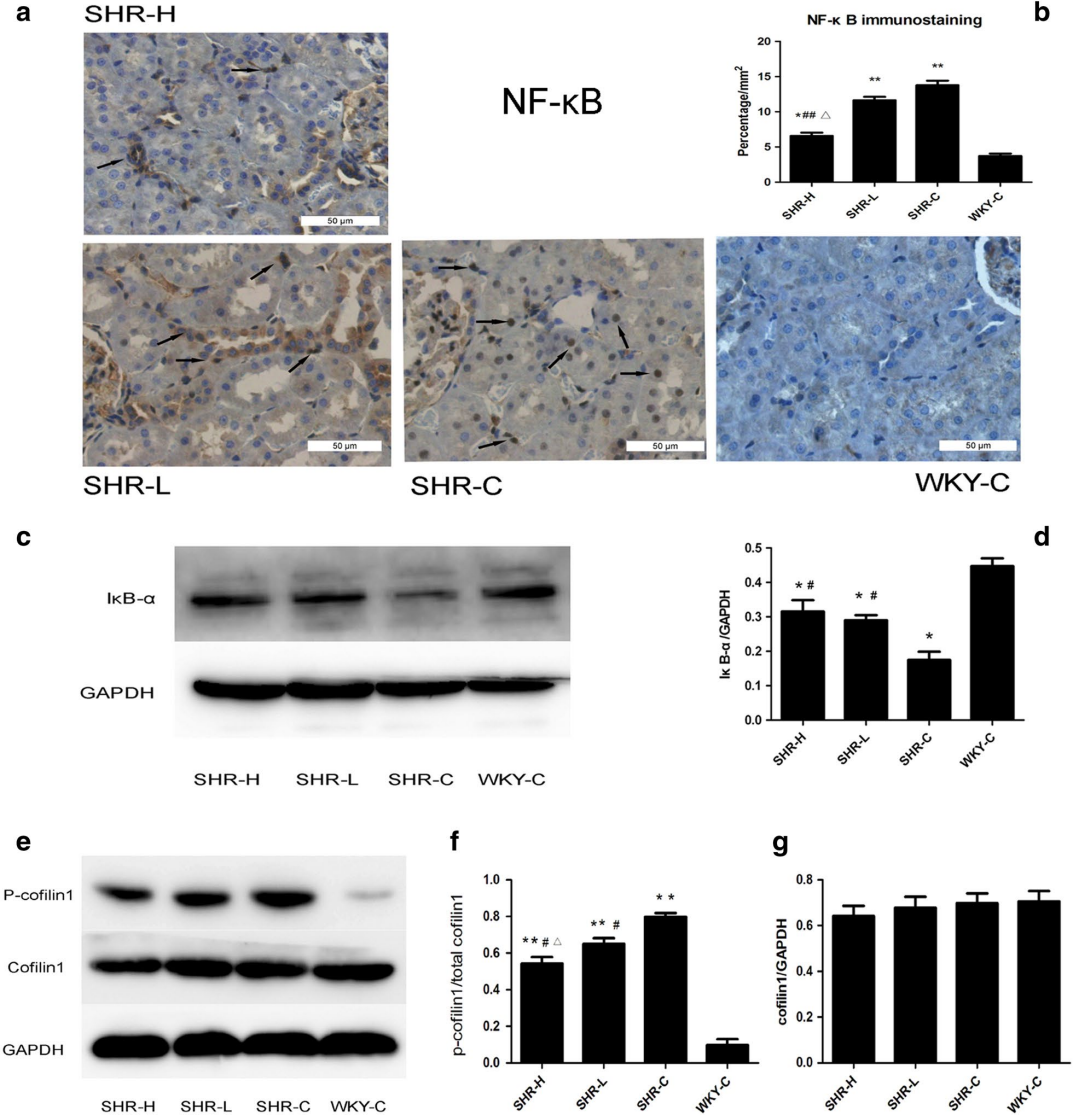
NF-κB localization and cofilin1 activity in the kidneys of SHRs. **a**, **b** NF-κB p65 location in different groups were detected by immunohistochemistry. Nuclear localization of NF-κB is highlighted by *arrows*. **c**, **d** The protein level of IκB-α in kidneys was detected by Western blot analysis. **e**–**g** The protein levels of cofilin1 and p-cofilin1 in the kidneys were detected by Western blotting analysis. Data are mean ± SD, n = 10. *P < 0.05, **P < 0.01 vs. WKY-C, ^#^P < 0.05, ^##^P < 0.01 vs. SHR-C and ΔP < 0.05 vs. SHR-L by ANOVA. Original magnification ×400, *Scar bar* represents 50 μm

Immunohistology staining for NF-κB p65 demonstrated that a small quantity of NF-κB p65 localized to the cytoplasm of the tubular epithelial cells in the WKY rats and that the nuclei were relatively unstained. By contrast, NF-κB p65 expression was increased in the cytoplasm and nuclei of the SHR kidneys. NF-κB p65 localized primarily to the nuclei of the renal tubular epithelial cells, a finding indicative of its increased activity, particularly in the SHR-C group. Treatment with high dose (250 mg kg^−1^ day) GSPE decreased NF-κB activity in SHR kidneys (P < 0.01, Fig. 2a, b); however, there was no significant difference between the SHR-L group and SHR-C group (P > 0.05). Furthermore, compared with the WKY rats, the protein level of IκB-α decreased in the kidneys of the SHR. However, following GSPE treatment, the decreased IκB-α level was partially restored (P < 0.05, Fig. 2c, d), confirming that the NF-κB pathway is activated in hypertensive kidneys and that GSPE may potentially suppress NF-κB pathway activation in the setting of hypertensive renal injury. Furthermore, we investigated cofilin1 activity by assessing the ratio of inactive cofilin1(phosphorylated cofilin1) to total cofilin1. As depicted in Fig. 2e–g, the protein content of p-cofilin1 was significantly increased in the SHRs, which indicates that the activity of cofilin1 was significantly decreased. Additionally, the activity of cofilin1 was significantly increased by GSPE treatment in a dose dependent manner (P < 0.05).

### The protein expression of MCP-1, IL-1β, cofilin1 and p-cofilin1 in AngII-induced HK2 cells

**Fig. 3:**
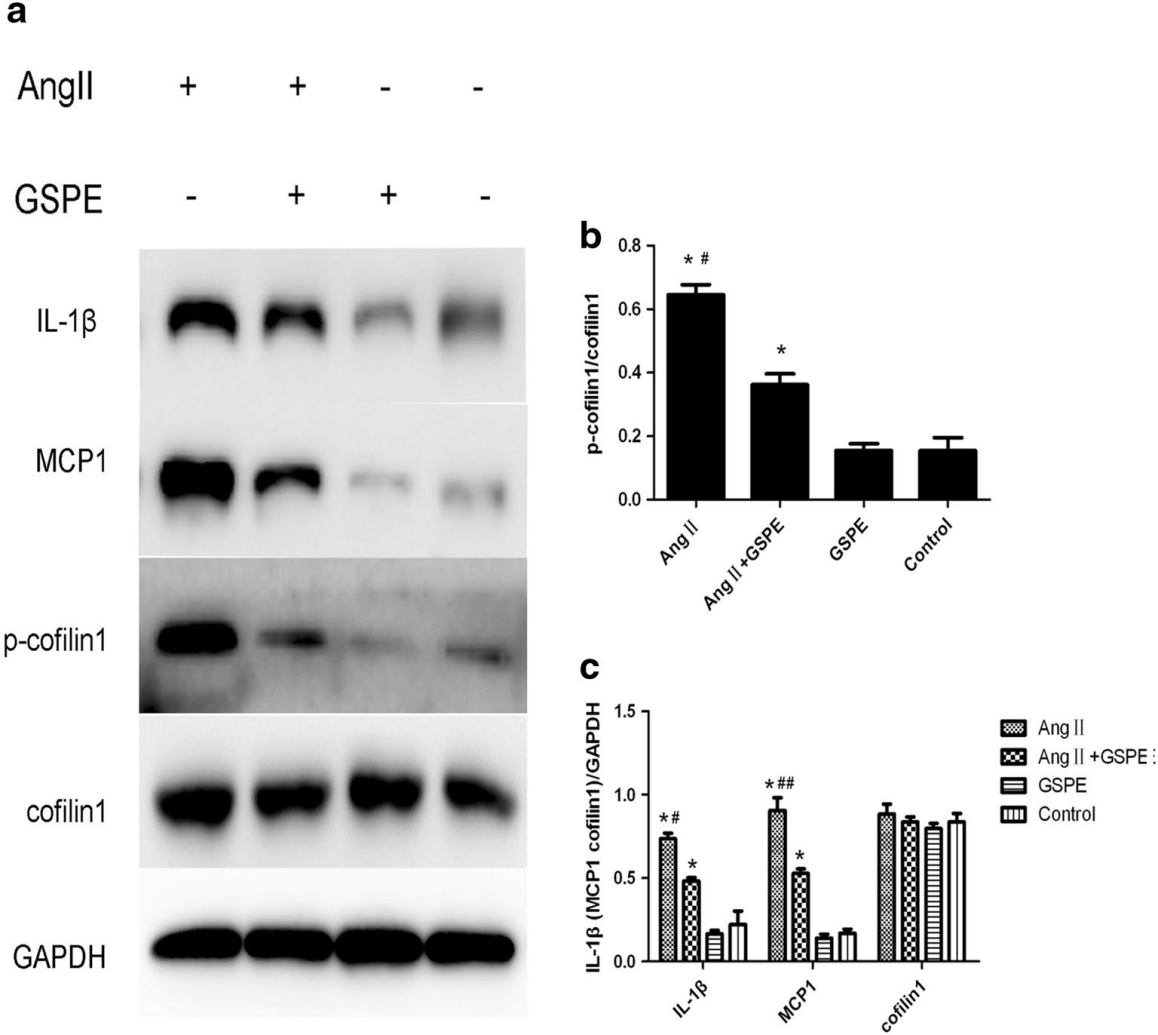
Inflammation and cofilin1 activity induced by Angiotensin II in the cultured HK-2 cells. HK-2 cells were pre-incubated with or without GSPE (50 μg mL^−1^) for 12 h and then treated with AngII 10^−6^mol l L^−1^ for 12 h. **a** Then total cell lysates were immunoblotted with anti-IL-1β, anti-MCP1, anti-p-cofilin1 and anti-cofilin1 antibodies. **b** Quantification of p-cofilin1 level normalized to cofilin1 in **a**, **c** quantification of IL-1β, MCP1, cofilin1 levels in **a**.  Values are mean ± SD, *P < 0.01 vs. control group, ^#^P < 0.05, ^##^P < 0.01 vs. GSPE + AngII treated group

Western blotting was performed to examine the protein expression of MCP1 and IL-1β in vitro. AngII treatment for 12 h significantly increased the protein levels of MCP-1 and IL-1β compared with the control group in the HK2 cells (P < 0.01). Additionally, stimulation with AngII promoted p-cofilin1 expression, resulting in cofilin1 inactivation. GSPE (50 µg mL^−1^) significantly reduced the levels of the above-mentioned proteins in the AngII-treated HK2 cells (P < 0.05, Fig. 3).

### The effects of cofilin1 knockdown on angiotensinII-induced stress fibre formation in HK2

**Fig. 4:**
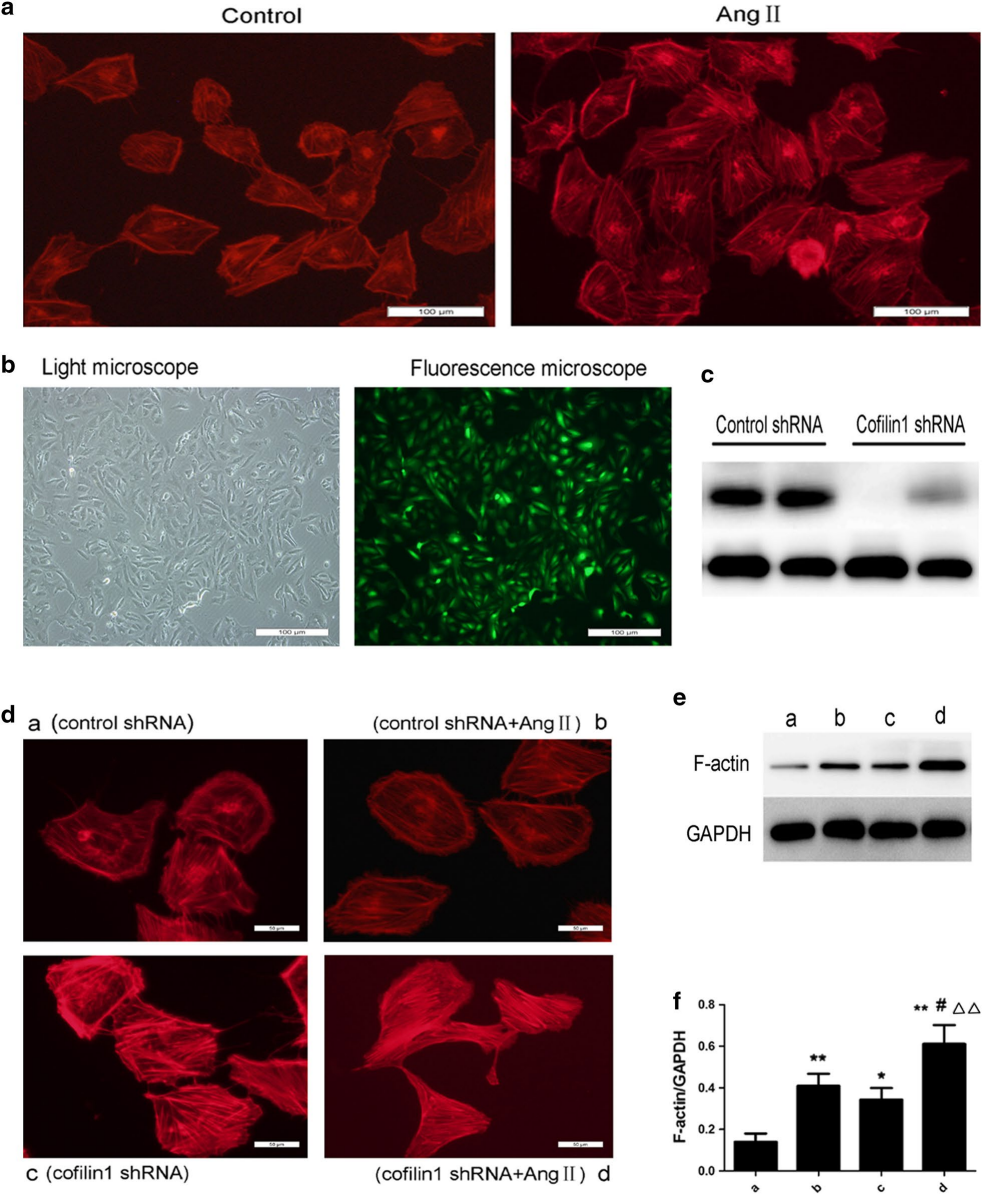
Effects of cofilin1 depletion on stress fibre formation. **a** AngII induced stress fibre formation in HK2. Confluent HK2 cells were untreated (*left*) or challenged for 12 h with AngII (*right*). The cells were then fixed, permeabilized, and stained with rhodamine-labeled phalloidin to visualize the actin stress fibres by fluorescence microscopy. Original magnification ×200, *scale bar* represents 100 μm. **b**, **c** Effect of lentiviruses transfection in HK2. The prepared HK2 cells were transfected with lentiviruses containing cofilin1 shRNA (Lv-cofilin1-shRNA) and nonspecific control lentiviruses (Lv-control-shRNA) at MOI of 50. **b** The infection efficiency was measured after 72 h using a fluorescence microscope via observing the expression of *green* fluorescent protein. Original magnification ×200, *scale bar* represents 100 μm. **c** Total transfected cell lysates were prepared and immunoblotted with an anti-cofilin1 antibody. **d**–**f** Cofilin-1 depletion promoted stress fibre formation. After passage, the cells transfected lentiviruses were challenged for 12 h with AngII (10^−6^mol L^−1^). **d** Stress fibre was visualized by fluorescence microscope. Original magnification ×400, *scale bar* represents 50 μm. **e**, **f** Representative Western blotting for F-actin, Cells in group *a*, *c* were only transfected with Lv-control-shRNA and Lv-cofilin1-shRNA respectively. Cells in group *b*, *d* were transfected with Lv-control-shRNA and Lv-cofilin1-shRNA respectively, then stimulated with AngII after transfection, *P < 0.05, **P < 0.01 vs. group *a*; ^#^P < 0.05 vs. group *b*; ΔΔP < 0.01 vs. group *c*

Rhodamine-labelled phalloidin staining was used to visualize the actin stress fibres via fluorescence microscopy and demonstrated that AngII stimulation increased stress fibre formation (Fig. 4a). To evaluate the effects exerted by cofilin1 on stress fibre formation, the cells were transfected with lentiviruses containing cofilin1 shRNA (Lv-cofilin1-shRNA) and non-silencing shRNA (Lv-control-shRNA). Transfection efficiency was assessed via fluorescence microscopy (Fig. 4b), and cofilin-1 expression was analysed via immunoblotting (Fig. 4c). The cells transfected with LV-cofilin-shRNA exhibited significantly decreased levels of cofilin-1 compared with the cells transfected with the LV-control-siRNA. We analysed different generations (band 3 is the second generation, whereas band 4 is the third generation) of cells transfected with LV-shRNA and used the second generation cells transfected with the lentivirus throughout the vitro experiments (Fig. 4c). We observed that the depletion of cofilin-1 increased F-actin expression and excessively augmented AngII-induced stress fibre formation (Fig. 4d–f), effects consistent with the actin depolymerising function of cofilin.

### The effects of cofilin1 knockdown on angiotensinII-induced NF-κB p65 nuclear translocation in HK2

**Fig. 5:**
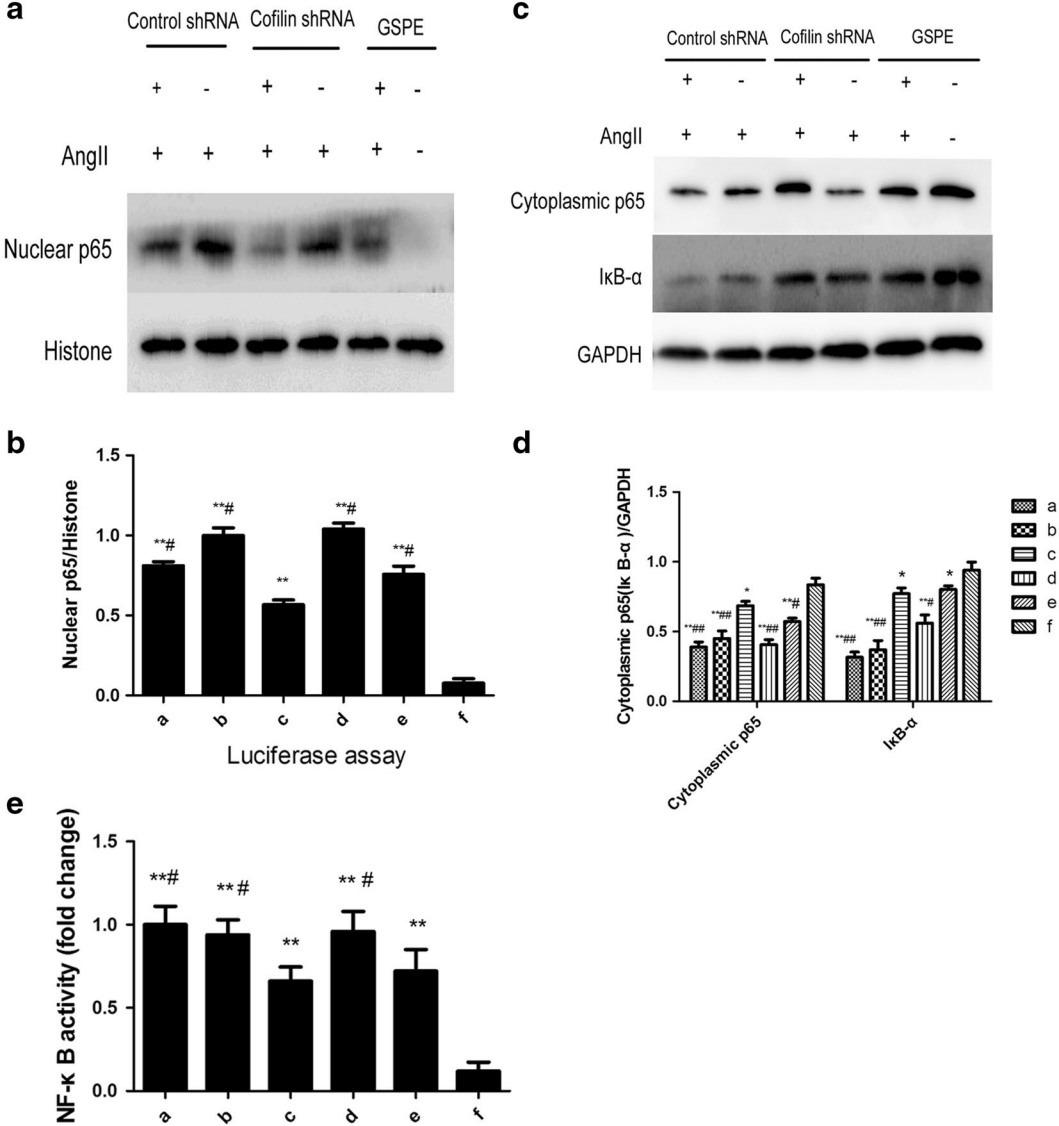
Effects of cofilin-1 knockdown on Angiotensin II-induced RelA/p65 nuclear translocation and NF-κB activity. After transfection and passage, cells were challenged for 12 h with AngII (10^−6^mol L^−1^). **a**–**d** Cytoplasmic and nuclear extracts were prepared and assayed for p65 and Iκ-Bα protein levels. *The bar graph* represents the effect of cofilin-1 depletion on AngII-induced p65 nuclear distribution and Iκ-Bα degradation. **e** Luciferase dual reporter gene assay of NF-κB activity. Cells in group *a*, *c* were transfected Lv-control-shRNA and Lv-cofilin1-shRNA respectively, then stimulated by AngII. Cells in *b* and *d* groups were stimulated by AngII without transfection. Cells in group **e** were preincubated with 50 μg mL^−1^ GSPE, then stimulated by AngII, while group *f* was normal control. The data are mean ± SD. *P < 0.05, **P < 0.01, vs. group *f*. ^#^P < 0.05, ^##^P < 0.01, vs. group *c*

**Fig. 6:**
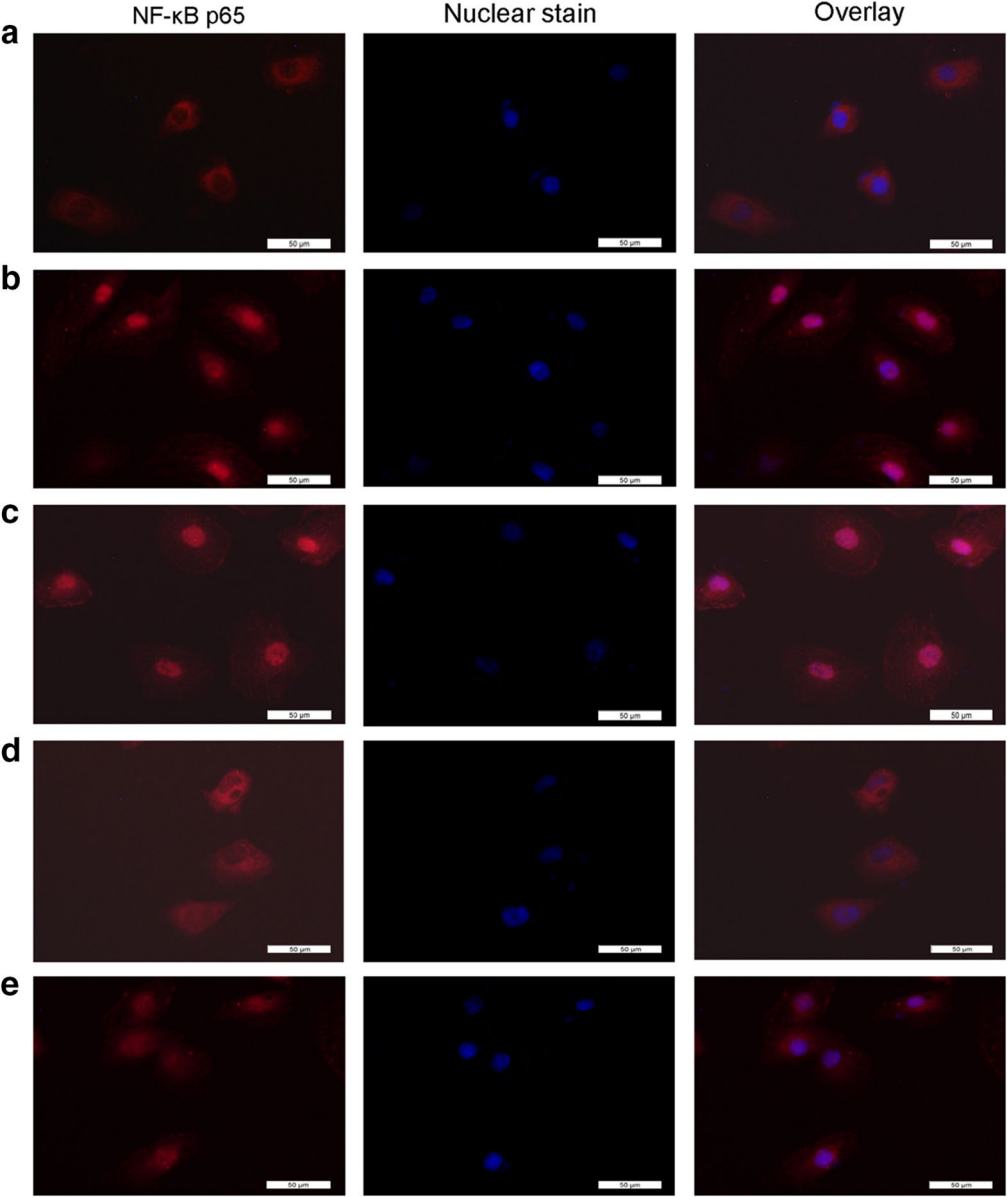
Immunofluorescence images of p65 nuclear translocation under the fluorescence microscopy. *Red* fluorescence indicates localization of NF-κB p65. Cofilinl knockdown suppressed AngII-induced nuclear translocation and activation of NF-κB: **a** without AngII stimulation NF-κB was predominately found in the cytoplasm. **b** With AngII stimulation, NF-κB translocated into the nucleus. **c** Transfection with nonspecific control lentiviruses had no effect on AngII-induced NF-κB translocation, as there were still obviously p65 staining in the nuclei. **d** Transfection with lentiviruses containing cofilin1 shRNA evidently inhibited the AngII-induced NF-κB translocation, as there was only a little of nuclear p65 staining found. **e** GSPE pretreatment inhibited AngII-induced NF-κB translocation. Magnification ×400. *Scale bar* represents 50 μm

An analysis of nuclear extracts via immunoblotting demonstrated that AngII -induced NF-κB p65 translocation into the nucleus was attenuated by either GSPE or cofilin1 depletion, which increased the level of NF-κB p65 in the cytoplasm (Fig. 5a–d). AngiotensinII stimulation resulted in reduced of IκB-α protein levels in the cytoplasm; however, said reduction was significantly reversed via either GSPE treatment or the shRNA knockdown of cofilin1 (P < 0.05, Fig. 5c, d). We subsequently examined NF-κB activity via a luciferase reporter assay, the results of which indicated that AngII stimulation resulted in increased NF-κB-dependent reporter activity, which was inhibited in the cells transfected with cofilin-shRNA or subjected to GSPE pretreatment(Fig. 5e). To confirm the suppressive effects of cofilin1 knockdown and GSPE on RelA/p65 nuclear accumulation, we investigated the distribution of NF-κB p65 via fluorescence microscopy (Fig. 6). NF-κB p65 was predominately located in the cytoplasm of the untreated cells, as expected. Stimulation with angiotensinII caused the translocation of RelA/p65 from the cytoplasm to the nucleus, irrespective of whether control shRNA was utilized. GSPE and the knockdown of cofilin1 decreased the distribution of NF-κB p65 staining in the nucleus, confirming that NF-κB p65 nuclear translocation is regulated by GSPE and cofilin1 in AngII-treated HK2 cells.

### The effects of cofilin1 knockdown on protein expressions of MCP-1, IL-1β in AngiotensinII-induced HK2 cells

**Fig. 7:**
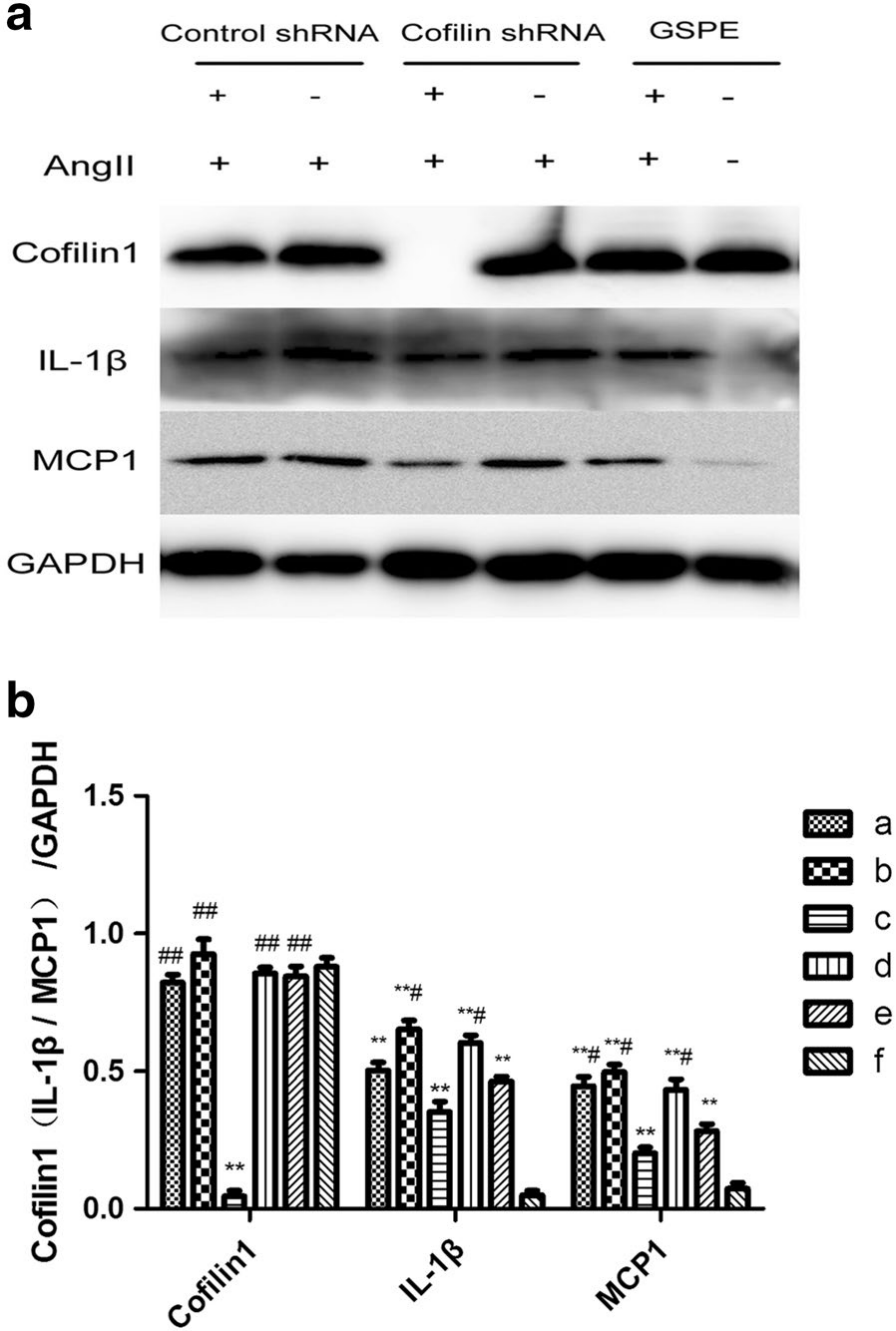
Effects of cofilin-1 knockdown on Angiotensin II-induced expression of inflammatory mediators. **a** After stimulation with AngII, cells extracts were prepared and assayed for protein levels of cofilin1, IL-1β, MCP1 by Western blotting. Grouping method is the same as Fig. 5. **b** Quantification of cofilin1, IL-1β, MCP1 levels in **a**. The data are the mean ± SD. *P < 0.05, **P < 0.01, vs. group *f*, ^#^P < 0.05, ^##^P < 0.01, vs. group *c*

AngII stimulation for 12 h significantly increased the levels of MCP-1 and IL-1β compared with the control group, as demonstrated via the Western blotting analysis. Cofilin1 deletion significantly reduced said protein levels in the AngII-treated HK2 cells (P < 0.05, Fig. 7).

## Discussion

The present study demonstrated that increased renal inflammation was accompanied by the inactivation of cofilin in middle-aged hypertensive rats. The depletion of cofilin1 excessively augmented stress fibre formation, caused the stabilization of actin filaments, and also impaired AngII-induced NF-κB activity in HK2 cells. Additionally, depleting cofilin1 inhibited the expression of both MCP1 and IL1β. These findings indicate that cofilin1 may be involved in hypertensive renal damage via the regulation of the nuclear translocation of NF-κB and inflammatory mediators.

The spontaneously hypertensive rat (SHR) is the most widely used animal model of human essential hypertension. Hypertensive kidney damage is not morphologically evident before 30 weeks of age in SHRs [17]. The damage then progresses slowly with increasing age, in a process similar to human hypertensive renal damage. In the present study, we selected 43-week-old SHRs as subjects, which have seldom been studied. Compared with age the matched WKY rats, more extensive tubulointerstitial inflammatory cell infiltration and tubular edema and increased 24-h urine protein levels were observed. Previous studies have observed that morphological renal injury of hypertension includes the following features: vasal intima and media hypertrophy, as well as necrosis, tubular atrophy and interstitial fibrosis, glomerular hypertrophy, sclerosis and proteinuria, and inflammation [18]. Therefore, we hypothesized that the renal damage observed in the 43-week-old SHRs represented a relatively early stage of the disease, in which inflammation plays an important part. Inflammatory cell infiltration is the result of the increased expression of inflammatory cytokines and chemokines, as demonstrated by the increased expression of inflammatory mediators such as MCP1 and IL-1β in the kidneys of SHRs. Numerous studies have demonstrated that MCP1 and IL-1β participate in different inflammatory states associated with renal diseases, including ischaemia/reperfusion injury [19], kidney transplantation [20], diabetic nephropathy [21], and hypertensive renal damage [22].

As inflammatory mediators MCP1 and IL-1β are both downstream products of activated NF-κB, we studied NF-κB activity in SHRs. As expected, our study demonstrated that the activity of NF-κB was significant increased in the middle-aged spontaneously hypertensive rats. In fact, NF-κB activity is upregulated in young normotensive SHRs and becomes more intense as the SHRs age and hypertension worsens [23]. To further elucidate the molecular mechanisms underlying hypertensive renal inflammation, we also performed in vitro research. AngII is involved in the pathogenesis of renal disease [24, 25] and contributes to the migration of inflammatory cells into the kidneys. Therefore, we studied the effects of AngII in HK2 cells. NF-κB p65 expression was significantly increased in the nuclei, and NF-κB activity was elevated in the AngII-induced HK2 cells. Moreover, both IL-1β and MCP1 were highly expressed in HK2 following AngII stimulation. Meanwhile the protein level of Iκ-Bα in the cytoplasm decreased both in the in vivo and in vitro study, a finding suggestive of the activation of the canonical NF-κB pathway in the setting of hypertensive nephropathy. This canonical pathway entails the activation of Iκ-Bα kinase and the subsequent phosphorylation-induced proteolysis of Iκ-Bα inhibitors, phosphorylation of the NF-κB p65 subunit and the nuclear translocation of the active NF-κB complexes, which act as transcription factors [26].

Our previous study demonstrated that cofilin1 is highly expressed in both the hearts and the arteries of SHRs via a comparative proteomics analysis (unpublished). Therefore, we studied the changes in cofilin1 expression in the setting of hypertensive renal damage and observed that there were no differences in the total cofilin1 protein content in the kidneys of the SHR and WKY rats; however, the phosphorylation of cofilin1 was significantly increased in the SHRs compared with the WKY rats. Regarding the in vitro study, in addition to the nuclear translocation of p65, stimulation with AngII simultaneously increased the phosphorylation of cofilin1, providing us with new insights into the relationship between NF-κB nuclear translocation and cofilin1 in HK2 cells.

Cofilin-1 is believed to play an essential role in RelA/p65 nuclear translocation regulation in thrombin-treated endothelial cells [8]. However, there are no reports regarding whether cofilin1 is involved in the AngII-induced nuclear translocation of NF-κB in HK2. To address this problem, cofilin1 was depleted using lentiviral-shRNA in the HK2 cells. Decreases of p65 nuclear translocation and NF-κB activity confirmed above-mentioned observations. However, the underlying molecular mechanisms of this phenomenon remain unknown. In response to specific stimuli, the translocation of NF-κB (p65) from the cytoplasm to the nucleus requires dynamic alterations of the actin cytoskeleton. Both the stabilization and destabilization of the actin cytoskeleton inhibit the nuclear accumulation of RelA/p65 [6]. Cofilin1 is a ubiquitous actin binding protein and is essential for actin filament elongation and remodelling. Activated cofilin1 regulates actin dynamics, either by depolymerising actin filaments at their pointed ends or by creating new filament barbed ends for F-actin assembly via their severing activity [27–29]. When cofilin is phosphorylated by either LIMK1/2 or TESK, it can no longer bind to either F-actin or G-actin [30]. Furthermore, cofilin1 is necessary for the maintenance of normal actin dynamics in renal cells such as podocytes and tubular epithelial cells [31, 32]. Taken together, these findings support the idea that cofilin1 may regulate the formation of stress fibre and also mediate the nuclear translocation of NF-κB. We observed that cofilin1 deletion resulted in the excessive augmentation of stress fibre formation, hindered NF-κB translocation and decreased the expression of its target proteins, MCP1 and IL1β in the HK2 cells.

It should be noted that some studies observed results different from ours. Németh et al. observed that the disruption of the actin cytoskeleton results in NF-κB activation and inflammatory mediator production in intestinal epithelial cells [33]. Kilian et al. demonstrated that Ang II induces cofilin dephosphorylation in tumour cells [34]. First, it has been reported that actin cytoskeleton-dependent and -independent pathways may facilitate RelA/p65 nuclear translocation in endothelial cells. The pathways associated with NF-κB activation respond to different stimuli [6]. We speculate that this phenomenon is also present in other cells, the activation pathways may be complementary. Second, it remains controversial which state (phosphorylated or non-phosphorylated) of cofilin1 predominates in the settings of various diseases. Our results were consistent with those of Zeidan [35], as we determined that Ang II induces the phosphorylation of cofilin1. Additionally, in both murine and human podocytes, PMA stimulation induces the activation of cofilin-1, whereas treatment with TGF-β results in cofilin-1 inactivation [31]. In summary, diverse cell types, stimuli and agents may result in differences between our studies and others.

An anti-inflammatory agent, GSPE was used in the positive control groups in the present study. A growing number of studies suggest that the dietary intake of GSPE is associated with both lower blood pressure and a reduced incidence of cardiovascular disease [36, 37]. However, the SHRs treated with GSPE for 22 weeks in our study exhibited decreases in only albuminuria; no discernible effects on blood pressure were noted. Significantly reduced inflammatory cell infiltration and MCP1 and IL-1β accumulation were also observed following GSPE administration in the kidneys of the SHRs. These reductions correlated positively with GSPE dosages. Furthermore, we observed that GSPE administration upregulated cofilin1 activity in the kidneys of the SHRs. GSPE also ameliorated inflammation and cofilin1 activity in AngII-induced HK2 cells. We observed that cofilin1 deletion and GSPE treatment play similar roles in the inhibition of NF-κB activity.

## Conclusion

Taken together, the present data suggest that cofilin1 is involved in hypertensive renal inflammation by regulating the nuclear translocation of RelA/p65 in renal tubular epithelial cells.

## Additional file

10.1186/s12967-015-0685-8 Time course studies of SBP in different groups over a twenty-two-week period. SBP was measured weekly using a tail-cuff non-invasive sphygmomanometer. In each group, n = 10. Data are shown as mean ± SD. SBP, systolic blood pressure. **P < 0.01 vs. WKY-C group.
